# Treatment of post-anesthesia dementia with perispinal etanercept injection and hyperbaric oxygen therapy: a case report

**DOI:** 10.1186/s13256-017-1259-6

**Published:** 2017-04-14

**Authors:** Steve Best, Dan G. Pavel

**Affiliations:** 1 The Neuroscience Center, 440 Lake Cook Road, Suite 2, Deerfield, IL 60015 USA; 2 PathFinder Brain SPECT, 440 Lake Cook Road, Suite 3, Deerfield, IL 60015 USA

**Keywords:** Dementia, Perispinal etanercept, HBOT, SPECT, Case report

## Abstract

**Background:**

We report on the first case of successful treatment for post-anesthesia dementia with perispinal etanercept injection combined with hyperbaric oxygen therapy.

**Case presentation:**

Our patient was a 77-year-old Caucasian man of Mexican ethnicity who presented to our clinic 4.5 years after a knee replacement surgery. Immediately post-surgery, the patient began to show dramatic cognitive, physical, and emotional impairment compared with his presurgical state; these symptoms were still present when he arrived at our clinic. A clinical assessment and brain single-photon emission computer tomography were performed. Diagnoses of dementia with major cognitive deficits and aphasia were established. A 40-session course of hyperbaric oxygen therapy was initiated to address our patient’s impairments. After the first ten hyperbaric oxygen therapy treatments, our patient was administered 25 mg perispinal etanercept injections approximately once weekly for 5 months. Starting after the first perispinal etanercept injection, our patient began showing progressive improvements. By the 5-month follow-up, his cognitive and physical function were substantially restored. A follow-up single-photon emission computer tomography scan showed increased perfusion in several small, localized areas.

**Conclusions:**

In this case of dementia and major cognitive disorder post major surgery and anesthesia, the very beneficial effect of combining hyperbaric oxygen therapy with perispinal etanercept is outlined.

## Background

We report on the first case, to the best of our knowledge, of the use of hyperbaric oxygen therapy (HBOT) followed by perispinal etanercept (PSE) injection to successfully treat dementia-like symptoms of sudden onset after orthopedic surgery with general anesthesia. Such symptoms can be related to pathologically prolonged microglial activation [1], which causes an excess of pro-inflammatory cytokine production, including tumor necrosis factor (TNF)-alpha. PSE injections have been postulated to reduce inflammatory activation by decreasing TNF-alpha bioactivity in the central nervous system [2–5]. Similarly, HBOT appears to have anti-inflammatory effects. It is believed to reduce pro-inflammatory cytokine activation production by means of provocation of stem cell activity [6–9].

We conceptualized the post-anesthesia cerebral insult, described in more detail below, as a type of head injury. Over the past several years, our clinic has seen positive results from using both PSE injection and HBOT, separately, for head injuries. PSE injection and HBOT were combined in the present case for two main reasons. The first was that we surmised the injury in the present case resulted from multiple causes—including consequences of surgery, anesthetic effects, and subclinical emboli—and wanted to provide a broader range of treatments to maximize the odds of successful response. Second, and related to the first point, we believed that the benefits of PSE injection and HBOT would be additive.

## Case presentation

A 77-year-old Caucasian man of Mexican ethnicity presented to our clinic 4.5 years after a knee replacement surgery. A retired repairman and avid cyclist, our patient had generally been in good health prior to the procedure. He was responsible for all his own daily activities such as feeding and bathing, cycled 25 miles per week, was an automobile enthusiast who went on regular walks to admire cars, and socialized with his family.

The patient’s family reported that they noticed marked changes in the patient starting immediately post-surgery. The night of the surgery, he became fearful he had been kidnapped and, in an attempt to escape, fell. He was unable by the next day to remember how to write his name. The family was assured by hospital staff that no adverse events had occurred that would impact the patient’s health. Nonetheless, over the following 3 months the patient continued to show dramatic cognitive, physical, and emotional impairment compared with his presurgical state. He could not recall his date of birth, sign his name, remember his children’s names, or find his way home from previously familiar locations. When spoken to, the patient appeared unable to comprehend language. He could not bathe himself, use the restroom, or eat on his own. He was unable to ride his bike, seeming unsure of the necessary movements. He slept through much of the day. At night, the patient could not sleep and would attempt to leave his home, although he was largely unable to walk. For the first time in his life, the patient showed high levels of anxiety.

Three months post-surgery, the patient was taken to a neurologist who diagnosed him with Alzheimer’s disease. The patient was started on 10 mg of diazepam and 13.3 mg of Exelon (rivastigmine) once daily. The medication sedated the patient during the day, although his insomnia persisted at night and the medication did not improve his cognitive abilities or activities of daily living (ADL). The patient’s primary care physician also concluded he had dementia and advised that little could be done to restore him to his previous level of functioning.

The patient’s symptoms/signs continued during the following years and were still present when he arrived at our clinic 4.5 years post-surgery. His medical history was notable for type 2 diabetes, hypertension, prostate hypertrophy with history of urine retention, arthritis, and obesity. His family history was positive for type 2 diabetes. His medication list included diazepam, Exelon (rivastigmine), Rapaflo (silodosin), atorvastatin, quetiapine, pantoprazole sodium, cephalexin, Janumet XL (sitagliptin and metformin hydrochloride), meclizine hydrochloride, Namenda (memantine), omega-3, metoprolol, ibuprofen, ramipril, glimepiride, and Lyrica (pregabalin). In view of his clinical status, a neuropsychologic testing could not be performed. He was essentially mute and disengaged from the clinical interview.

**Fig. 1:**
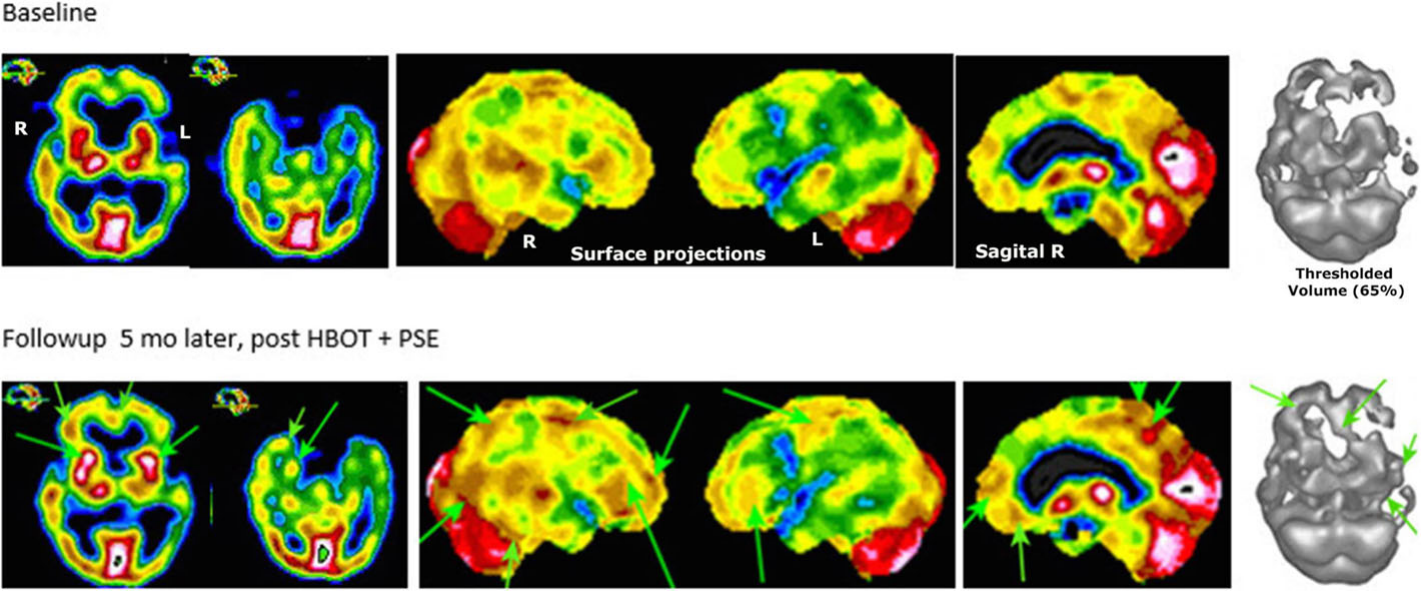
Single-photon emission computed tomography imaging results. The *color code* indicates the level of blood flow, which in turn is proportional to the metabolism level. This enables the detection of hyper or hypo functioning areas: from lowest levels (*blue hues*) to highest levels (*white and black surrounded by white*). The black and white display is a volumetric rendition at a standardized threshold level for both studies. *Upper row*: a baseline single-photon emission computed tomography image revealed extensive left hemispheric underperfusion, and multiple localized underperfusions in the right hemisphere. Bilateral involvement of parts of the dorsolateral prefrontal cortex, and extensive underperfusion in the temporal lobes (more on the left) and to a lesser extent in the orbitofrontal areas. *Lower row*: a follow-up single-photon emission computed tomography image after 5 months showed similar overall appearance but with increased perfusion in multiple small localized areas namely in parts of the anterior aspect of the prefrontal cortex (including in the ventromesial aspect), right superior parietal, right lateral occipital, superior aspect of the left frontoparietal area, posterior cingulate/precuneus and apico-mesial right temporal. In addition, significant increase was seen in the striatum bilaterally. *HBOT* hyperbaric oxygen therapy, *PSE* perispinal etanercept

We performed a baseline single-photon emission computer tomography (SPECT) functional imaging with 99 m-Tc HMPAO. Brain SPECT is a neuroimaging technique that reveals the functional status of gray mater areas via relative perfusion three-dimensional mapping [10–13]. Results from this initial assessment (see upper row of Fig. 1) showed extensive left hemispheric underperfusion, and multiple localized underperfusions in the right hemisphere. On both sides there was also involvement of the dorsolateral prefrontal cortex (DLPFC), and extensive underperfusion in the temporal lobes (primarily on the left) and to a lesser extent in the orbitofrontal areas. Such a combination of abnormal areas can be seen in major cognitive dysfunctions.

Concerning the etiology of our patient’s symptoms, while a component of degeneration (Alzheimer’s disease) could be considered, this alone was not justified by the SPECT scan. A main reason is that the posterior cingulate/precuneus area would have been expected to be definitely abnormal, but there was only very limited underperfusion in that region. The possibility of superimposed vascular etiology therefore had to be considered. Based on our patient’s medical history and SPECT results, we hypothesized that he had experienced a cerebral insult during his knee surgery, namely that the injury resulted from multiple causes, including consequences of surgery, and anesthetic effects [14, 15] that created perfusion abnormalities simulating those observed after stroke. To address these cognitive, physical, and emotional impairments, a treatment plan was prepared including both HBOT and PSE administration.^1^ The rationale was that our patient’s clinical status was so abnormal that multiple ways to reduce inflammation and increase stem cell activity were needed as soon as possible. Additionally, based on previous experience related to the treatment of head injuries separately with HBOT and PSE injections, we believed that their effect would be additive. Based on our experience over the past 15 years a 40-session course of HBOT was initiated. Indeed it is known from the treatment of traumatic brain injury (TBI) that for significant improvements to occur, a minimum of 20 sessions is needed. In his case the damage was so extensive that 40 sessions were considered as a necessity. The treatments were administered daily in a multiplace chamber for 60 minutes at a depth of 1.75 atmosphere absolute (ATA). After the first ten HBOT treatments, we began administering a 25-mg PSE injection approximately once weekly for 5 months. Before starting PSE, the patient had a purified protein derivative (PPD) test, which was negative. The 20 injections were made subsequent to HBOT sessions during the lengthy duration of the HBOT treatment and beyond, at 1-week intervals for 5 months. The number of injections was higher than in most patients based on the same consideration as above and on our experience of 4 years with PSE.

Concerning both treatments, based on literature and on our experience, there are very rare side effects for either procedure and when they occur are easily treated and reversible.

Our patient began showing progressive improvements starting after the first PSE injection. By the 5-month follow-up, his cognitive functioning had been substantially restored. He remembered his own name and those of his children. Although he was still not able to speak as well as he could presurgery, he returned to socializing with his family and was able to clearly understand and engage in conversation, for example laughing at a joke or telling a family member to “calm down” when upset. Perhaps even more remarkably, our patient’s physical functioning improved. We view this as consistent with the hypothesis that his symptoms reflected an acute event that left dormant but preserved viable tissue surrounding the main affected areas, as opposed to a degenerative disease. Our patient improved to the point of being able to completely care for himself, including feeding and bathing. He could go for walks on his own without getting lost, during which he showed interest in cars as he had before, and his premorbid personality style returned. A healthy sleep pattern was largely restored, with our patient sleeping about 7 hours each night. This was in stark contrast with his complete inability, when he first presented, to care for himself or interact with others. Because no neuropsychologic evaluation could be done originally, we considered that there was no point doing so at this time. His relatives were elated that he was now able to return to his place in the family dynamics.

A follow-up brain SPECT scan was done 5 months after the baseline study. While the overall appearance looked similar to the first scan, we nonetheless found increased perfusion in multiple small, localized areas in cortical and subcortical structures (see the lower row and green arrows in Fig. 1). Among them were parts of the anterior aspect of the prefrontal cortex (including the ventromesial aspect), right superior parietal, right lateral occipital, superior aspect of the left frontoparietal area, posterior cingulate/precuneus and apico-mesial aspect of the right temporal lobe. In addition, there was a significant perfusion increase in the striatum bilaterally.

At this point in time our patient is at 16 months since the end of the combined treatment and all the clinical improvements mentioned above are still maintained.

## Discussion

In this case report, the presence of chronic marked cognitive, physical, and emotional disability following a surgical procedure was supported by our evaluation at 4.5 years post-onset and by the functional brain SPECT imaging done at the same time. The fact that degeneration alone was not compatible with our evaluation suggested that there were still areas of gray matter underperfusion with sufficient viability. The concurrent treatment with PSE and HBOT produced early signs of improvement, which then continued during the period of 5 months and till now. This indicated that indeed the hypothesis of persisting viability in some areas was plausible.

As can be expected from a chronic condition, whatever its etiology, there were persistent areas of extensive abnormal perfusion and thus of metabolic abnormality. Nonetheless, the improvement in small but multiple areas of the gray matter, as documented by functional imaging, was enough to provide a major quality of life improvement. Indeed while a follow-up brain SPECT scan was still abnormal, there was evidence for improved perfusion in small key areas, specifically the mesial temporal lobe, prefrontal cortex, ventromesial frontal, posterior cingulate and precuneus areas, dorsal parietal and lateral occipital areas. Such areas are known to contribute to memory, cognition and behavior. The clinical improvement was also repeatedly confirmed by statements from his family, who greatly appreciated our patient’s restored independence and social life. A very recent follow-up showed that the same level of improvements is still present 16 months after the end of treatment.

## Conclusion

In this special case of dementia, with prolonged major cognitive deficits and aphasia, occurring post major surgery and anesthesia, the very beneficial effect of combining HBOT with PSE treatment is outlined.

## Abbreviations

ATA: Atmosphere absolute
DLPFC: Dorsolateral prefrontal cortex
HBOT: Hyperbaric oxygen therapy
PPD: Purified protein derivative
PSE: Perispinal etanercept
SPECT: Single-photon emission computer tomography
TBI: Traumatic brain injury
TNF: Tumor necrosis factor

## Footnotes

1 The method of perispinal administration of etanercept utilized was used under license from the patent holder, TACT IP, LLC, Boca Raton, FL, USA.

## References

[1] Terrando N, *et al.* Tumor necrosis factor-alpha triggers a cytokine cascade yielding postoperative cognitive decline. Proc Natl Acad Sci U S A. 2010;107:20518–22. 10.1073/pnas.1014557107 PMC2996666 21041647

[2] Clark IA, Vissel B. A neurologist’s guide to TNF biology, and to the principles behind the therapeutic removal of excess TNF in disease. Neural Plast. 2015;2015:358263. 10.1155/2015/358263 PMC4510439 26221543

[3] Siniscalchi A, *et al.* Anti-inflammatory strategies in stroke: a potential therapeutic target. Curr Vasc Pharmacol. 2016;14:98–105. 10.2174/1570161113666150923111329 26411421

[4] Tobinick E. Perispinal etanercept: a new therapeutic paradigm in neurology. Expert Rev Neurother. 2010;10:985–1002. 10.1586/ern.10.52 20518613

[5] Tobinick E, *et al.* Immediate neurological recovery following perispinal etanercept years after brain injury. Clin Drug Investig. 2014;34:361–6. 10.1007/s40261-014-0186-1 24647830

[6] Vlodavsky E, Palzur E, Soustiel JF. Hyperbaric oxygen therapy reduces neuroinflammation and expression of matrix metalloproteinase-9 in the rat model of traumatic brain injury. Neuropathol Appl Neurobiol. 2006;32:40–50. 10.1111/j.1365-2990.2005.00698.x 16409552

[7] Milovanova TN, *et al.* Hyperbaric oxygen stimulates vasculogenic stem cell growth and differentiation in vivo. J Appl Physiol. 2009;106:711–28. 10.1152/japplphysiol.91054.2008 PMC2644249 19023021

[8] Heyboer MI, *et al.* CD34+/CD45-dim stem cell mobilization by hyperbaric oxygen-Changes with oxygen dosage. Stem Cell Res. 2014;12:638–45. 10.1016/j.scr.2014.02.005 PMC4037447 24642336

[9] Li F, *et al.* Hyperbaric oxygenation therapy alleviates chronic constrictive injury-induced neuropathic pain and reduces tumor necrosis factor alpha production. Anesth Analg. 2011;113:626–33. 10.1213/ANE.0b013e31821f9544 21596875

[10] Catafau AM. Brain SPECT in clinical practice. Part I: perfusion. J Nucl Med. 2001;42(2):259–71. 11216525

[11] Holman BL, Devous Sr MD. Functional brain SPECT: the emergence of a powerful clinical method. J Nucl Med. 1992;33(10):1888–904. 1403163

[12] Minoshima S, *et al.* Anatomical standardization: linear scaling and nonlinear warping of functional brain images. J Nucl Med. 1994;35(9):1528–37. 8071705

[13] Juni JE, *et al.* Procedure guidelines for brain perfusion SPECT using 99mTc radiopharmaceuticals 3.0. J Nucl Med Technol. 2009;37(3):191–5. 10.2967/jnmt.109.067850 19692453

[14] Papon MA, *et al.* Alzheimer’s disease and anesthesia. Front Neurosci. 2011;4:272. 10.3389/fnins.2010.00272 PMC3034231 21344011

[15] Perouansky M, Hemmings HC. Neurotoxicity of general anesthetics: cause for concern? Anesthesiology. 2009;111:1365–71. 10.1097/ALN.0b013e3181bf1d61 PMC2784653 19934883

